# Supplementary material for: Exosomal Prostate-Specific Membrane Antigen (PSMA) and Caveolin-1 as Potential Biomarkers of Prostate Cancer—Evidence from Serbian Population
Source: Int J Mol Sci. 2024 Mar 21;25(6):3533. doi: 10.3390/ijms25063533 (PMC10970688; doi:10.3390/ijms25063533)

Supplementary Figure S1. Detection of EV surface markers in isolates from PCa (upper plane) and BPH (lower plane) subjects by flow cytometry. Isolated EXOs were immobilized on latex beads, beads were than treated with glycine and milk for blocking after with the surface markers were detected by flow cytometry using Anti CD9-PE. Latex beads without EXOs treated with glycine and milk are used as a control. Ctrl- marks control beas, S1-S5-mark samples from PCa and BPH subjects

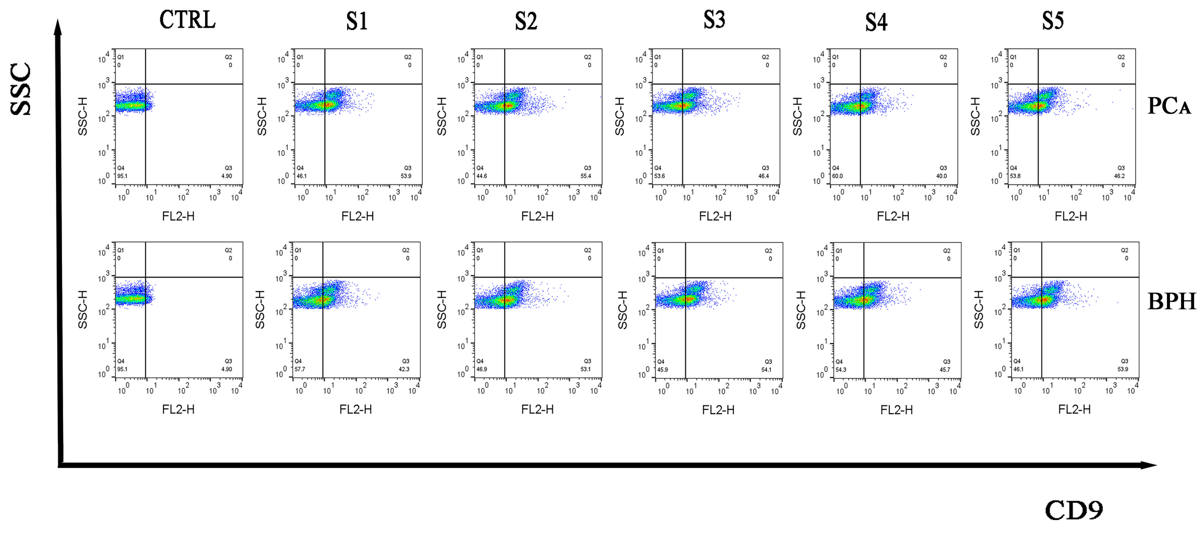

Supplement: Supplementary file 1 [file ijms-25-03533-s001.zip › ijms-2876704-supplementary.pdf]
